# Reducing Educational Bias in Cognitive Assessment via Dynamic Support Vector Machine Weighting: Validation Study on an Education-Stratified Dataset

**DOI:** 10.2196/79841

**Published:** 2026-02-25

**Authors:** Qing Liu, Chi Ma, Mengyuan Liu, Suhui Chen, Mengting Yu, Lijuan Xia, Qi Zhang, Ming Wu

**Affiliations:** 1 School of Humanities and Social Sciences, University of Science and Technology of China, Hefei, China; 2 School of the Gifted Young, University of Science and Technology of China, Hefei, China; 3 Department of Rehabilitation Medicine, The First Affiliated Hospital of USTC, Division of Life Sciences and Medicine, University of Science and Technology of China, Tian'e Hu No.1, Hefei, 230000, China, 86 186 5510 6697; 4 Department of Rehabilitation Medicine, The Second People's Hospital, Wuhu, China; 5 Shuguang Hospital Anhui Branch Affiliated to Shanghai University of Traditional Chinese Medicine, Hefei, China

**Keywords:** machine learning, Mini-Mental State Examination, MMSE, support vector machine, SVM, dynamic weighted model, educational background

## Abstract

**Background:**

The Mini-Mental State Examination (MMSE) remains widely used for cognitive screening, yet its performance varies substantially across educational backgrounds. Linear education corrections fail to capture the nonlinear interference patterns among subitems.

**Objective:**

This study aimed to analyze how educational level shapes MMSE subitem contributions and to develop an education-adaptive optimization strategy using support vector machine–based weighting.

**Methods:**

MMSE data from 812 participants were stratified into 4 education groups. Subitem deletion experiments quantified each subitem’s contribution (Δ). Education-specific support vector machine models were then constructed to derive dynamic weighting coefficients. Performance improvements were assessed before and after weighting.

**Results:**

The illiterate group relied heavily on spatial orientation and memory, whereas university-educated individuals depended more on executive and calculation functions. Several education-dependent interference items were identified (eg, visuospatial construction in the primary group and basic orientation tasks in the university group). Dynamic weighting improved accuracy in all cohorts, most notably among illiterate individuals (Δ*=*7.25%; *P*=.06), followed by the primary school group (Δ=3.12%; *P*=.03).

**Conclusions:**

Education-stratified weighting enhances the fairness and interpretability of MMSE-based screening. External validation confirmed generalizability, although multicenter studies are needed.

## Introduction

The Mini-Mental State Examination (MMSE) is one of the most widely used cognitive screening tools in clinical and community settings, designed for the rapid detection of cognitive impairment [1]. Since its development by Folstein et al in 1975 [2], the MMSE has become a core instrument for assessing five cognitive domains—orientation, immediate recall, attention or calculation, language, and visuospatial ability—and can typically be completed within 10 to 15 minutes [3]. Its broad adoption reflects its operational simplicity and solid psychometric performance, with reported sensitivity of 80% to 85% and specificity of 75% to 80% for dementia screening [4].

Alzheimer disease is characterized by progressive neurodegeneration, including amyloid-β deposition and structural decline. As Alzheimer disease progresses in a relatively predictable neuroanatomical sequence, MMSE subdomains provide clinically meaningful stage markers: orientation and memory deficits often correspond to early hippocampal involvement, while language and visuospatial impairments reflect later temporoparietal degeneration [5,6]. This multidimensional structure allows the MMSE to map aspects of the disease trajectory beyond a single total score.

Nevertheless, the diagnostic performance of the MMSE varies considerably across educational levels [7,8]. Individuals with higher education often exhibit false-negative results due to two well-recognized mechanisms: compensatory neuroplasticity that delays the manifestation of cognitive symptoms [9] and the use of test-taking strategies that allow them to maintain near-normal scores despite underlying pathology [10,11]. In contrast, individuals with limited formal education show substantially higher false-positive rates, particularly on education-dependent items, such as literacy tasks and object naming, with error rates increasing by approximately 30% to 35% compared with education-neutral instruments [12-15]. As a result, the MMSE demonstrates reduced sensitivity (68%‐72%) and specificity (65%‐70%) when a conventional cutoff score of 24 points is applied across heterogeneous educational backgrounds [16-18]. Current clinical guidelines therefore emphasize the need to incorporate educational history into cognitive assessment to minimize diagnostic inaccuracies [19,20].

Machine learning (ML) has emerged as a transformative approach in medical diagnostics, with particular relevance to cognitive assessment optimization. Support Vector Machines (SVMs) offer several advantages for psychometric refinement, including (1) robust performance in limited-sample settings (typically n<1000) [21,22], (2) effective handling and interpretability of high-dimensional feature spaces [23,24], and (3) strong compatibility with multimodal data integration frameworks that combine neuropsychological assessments with imaging- or biomarker-derived measures [25-28]. Earlier ML classifiers trained solely on MMSE total scores or subitems—most commonly logistic regression, SVM, or random forest—have demonstrated moderate diagnostic performance, with reported accuracies of 72% to 85% and area under the curve (AUC) values of 0.75 to 0.85 [29]. In contrast, multimodal models integrating magnetic resonance imaging (MRI) radiomics, positron emission tomography (PET) signatures, or speech biomarkers consistently achieve accuracies exceeding 90% to 95% [30,31]. This performance gap highlights the need for methodological innovations that enhance unimodal MMSE-based classifiers while preserving their scalability and low implementation cost.

This study introduces an education-sensitive adaptation of the MMSE based on an SVM-guided dynamic weighting framework. We analyzed cognitive screening data (n=812) collected from Chinese tertiary hospitals and community health centers and stratified participants into four educational cohorts: illiterate (0 y), primary (≤6 y), secondary (7‐12 y), and tertiary (≥13 y). The model uses systematic item-response analysis combined with SVM-guided weighting to adjust the relative contribution of each MMSE subitem in an education-specific manner.

This work provides 2 methodological innovations. First, it moves beyond conventional linear education corrections by using nonlinear SVM modeling to identify “cognitive interference” patterns unique to each education level. Second, it introduces a dynamic weighting strategy specifically designed to mitigate the disproportionate false-positive burden experienced by low-literacy populations. Together, these innovations aim to deliver a more equitable and clinically practical adaptation of the MMSE, particularly relevant for resource-limited settings.

Although unimodal or condensed classifiers derived solely from MMSE subitems are highly accessible, they are inherently constrained by limited feature richness and therefore tend to exhibit moderate predictive performance. In contrast, multimodal diagnostic systems—such as those leveraging MRI, PET, radiomics, or speech biomarkers—achieve substantially higher accuracies but remain impractical for widespread screening due to cost, technical requirements, and limited availability in primary care. Thus, methodological advances that enhance the diagnostic utility of unimodal MMSE-based approaches, while maintaining their affordability and scalability, are urgently needed. This study addresses this gap by proposing an education-stratified dynamic weighting method designed to improve predictive performance within the intrinsic limitations of unimodal cognitive classifiers.

## Methods

### Ethical Considerations

This study followed the principles of the Declaration of Helsinki and was approved by the Ethics Committee of the First Affiliated Hospital of the University of Science and Technology of China (approval number 2024-RE-431). All clinical and community datasets were obtained with authorization from the original data custodians. During data processing, only anonymized records were used, and no personally identifiable information (eg, names, addresses) was accessed, ensuring compliance with the Declaration of Helsinki.

### Sample

Cognitive assessment data were obtained from two sources: the Rehabilitation Medicine Department of a tertiary hospital and a community-based cognitive screening program. A total of 812 valid records were included. Among these, 404 samples were collected from outpatients and inpatients with clinically diagnosed cognitive impairment. The inclusion and exclusion criteria are summarized in Table 1.

**Table 1.** Data inclusion and exclusion criteria.

Source	Number (n)	Inclusion criteria	Exclusion criteria	Data assessment requirements
Clinical dataset	404	Outpatients and inpatients with clinically confirmed cognitive impairment Complete clinical and assessment records	Patients who underwent surgery or died during hospitalization Missing MMSE^a^ assessment data	For clinical data: All MMSE assessors completed a standardized 12-h training program and passed certification. Postcollection, automated missing value detection and manual verification were performed.
Community dataset	408	Community residents with no medical consultation for cognitive complaints within the past 12 mo Intact consciousness and ability to complete cognitive assessments	Individuals with diagnosed mild cognitive impairment or suspected cognitive decline Individuals with incomplete assessment procedures or severe organic neurological conditions	For community data: Assessments followed standardized community screening procedures. Assessments were administered following standardized community screening procedures with verification of data completeness and adherence to protocol.

a MMSE: Mini-Mental State Examination.

### Scale Selection and Scoring Criteria

The MMSE was used as the primary cognitive assessment tool. The scale comprises five cognitive domains: orientation, memory, attention and calculation, language, and visuospatial construction. Education-adjusted cutoff scores were based on Chinese normative standards [3]: illiterate group: ≤17, primary school group: ≤20, secondary school group: ≤22, and university group: ≤23.

### Data Processing and Grouping

#### Overview

A total of 1000 assessments were collected initially. After quality control, 188 (18.8%) records were excluded due to missing data (n=107, 10.7%), nonstandardized test administration (n=35, 3.5%), or unqualified assessors (n=46, 4.6%). Before merging the hospital and community datasets, baseline demographic comparability was examined using an independent-samples *t* test (age) and chi-square test (gender) to minimize potential selection bias.

The final dataset (N=812) was stratified by educational attainment as follows: illiterate (n=108, 13.3%), primary school (n=105, 12.9%), secondary school (n=364, 44.8%), and university (n=235, 28.9%). Within each group, patient and control counts were recorded as illiterate (patients: n=60, 7.4%; and controls: n=48, 5.9%), primary (patients: n=53, 6.5%; and controls: n=52, 6.4%), secondary (patients: n=185, 22.8%; and controls: n=179, 22.0%), and university (patients: n=106, 13.1%; and controls: n=129, 15.9%). This preprocessing ensured a standardized data foundation for subsequent ML procedures.

#### Model Selection

A SVM classifier with a Radial Basis Function kernel was selected due to its suitability for medium-sized datasets and its capacity to model nonlinear decision boundaries. A stratified 5-fold cross-validation scheme was used to maximize data utilization while preserving the original case-control ratio within each educational group.

#### Hyperparameter Optimization

Hyperparameters were optimized through a grid search embedded within the cross-validation framework. The search grid included penalty parameter C: {0.1, 1, 5, 10, 100} and kernel coefficient γ: {0.001, 0.01, 0.1, 1}.

For each parameter pair, the model was trained on 4 folds and evaluated on the remaining fold, and the average accuracy across all 5 folds was used to identify the optimal configuration. Education-specific models were then retrained on the full dataset using the selected hyperparameters to ensure maximal predictive performance while avoiding information leakage.

### Experimental Design

**Figure 1.**
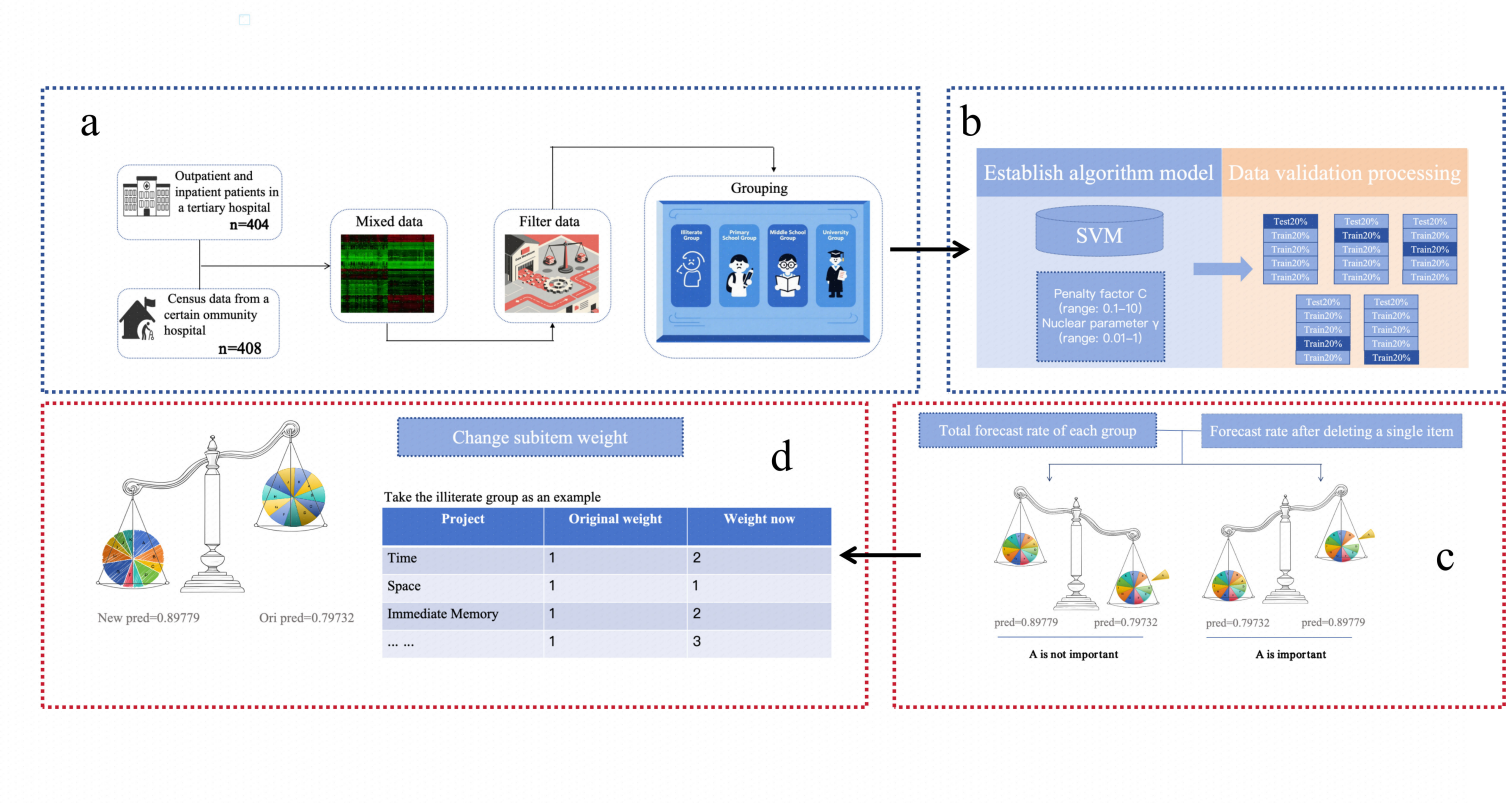
Contribution of individual Mini-Mental State Examination (MMSE)j items across educational strata. Feature importance was quantified using the percentage change in cross-validated accuracy (Δ_i_) after deleting each subitem. We operationally defined critical factors as items whose removal caused a performance decrease of *Δ*<−1.0 and interference factors as items whose removal increased accuracy by Δ＞+0.5. SVM: support vector machine.

The overall experimental workflow is presented in Figure 1.

The process began with data fusion and initial grouping: clinical and community data were merged to construct the initial dataset (n=812), and participants were stratified into 4 educational groups (ie, illiterate, primary school, secondary school, and university) to ensure subsequent analyses reflected education-specific cognitive characteristics. Next, a Support Vector Machine classifier with a radial basis function kernel was trained using a stratified 5-fold cross-validation scheme, preserving the original case-control ratio within each group. To quantify the contribution of each MMSE subitem, a systematic ablation procedure was performed. For each educational group, each subitem was removed in turn, and an SVM model was retrained. The feature contribution was quantified using the cross-validated change in prediction accuracy (Δ), calculated as:


$$\Delta =Accuracy_{postdeletion}-Accuracy_{baseline}$$

A negative Δ indicates a positive contribution (performance dropped when the item was removed), while a positive Δ suggests the item acted as noise. Finally, based on the subitem contribution profiles, an education-specific dynamic weighting scheme was constructed to adjust the relative importance of MMSE subitems before classification.

### Other Statistical Techniques

Routine statistical analyses were performed using SPSS version 24.0 (IBM Corp), and ML model construction and evaluation were conducted using Python (version 3.10.10; Python Software Foundation)

For categorical variables, chi-square or Fisher exact tests were applied depending on expected frequencies. Continuous variables with a normal distribution were expressed as mean (SD) and compared using independent-samples *t* tests. Variables not following a normal distribution were summarized using median (IQR) and analyzed using nonparametric tests.

A 2-tailed *P*<.05 was considered statistically significant.

## Results

### Demographic Comparison

Following data cleansing, demographic characteristics of the clinical and community datasets were compared to verify baseline homogeneity before integration. As presented in Table 2, differences in age and gender distributions were not statistically significant (*P*>.05).

**Table 2.** Demographic characteristics and data composition stratified by educational background.

Characteristic	Illiterate (n=108)	Primary school (n=105)	Secondary school (n=364)	University (n=235)	*P* value
Age, mean (SD)	59.02 (8.5)	58.55 (7.8)	58.62 (8.1)	56.7 (7.4)	.05
Gender, n (%)					.37
Male	67 (62.0)	60 (57.1)	219 (60.2)	140 (59.6)	
Female	41 (38.0)	45 (42.9)	145 (39.8)	95 (40.4)	
Data source, n (%)					**.**12
Clinical^a^ (patient)	60 (55.6)	53 (50.5)	185 (50.8)	106 (45.1)	
Community^b^ (control)	48 (44.4)	52 (49.5)	179 (49.2)	129 (54.9)	

a Hospital dataset: patients diagnosed or evaluated in the tertiary hospital.

b Community dataset: participants recruited from community-based cognitive screening programs.

### Item-Wise Contribution Analysis

**Figure 2.**
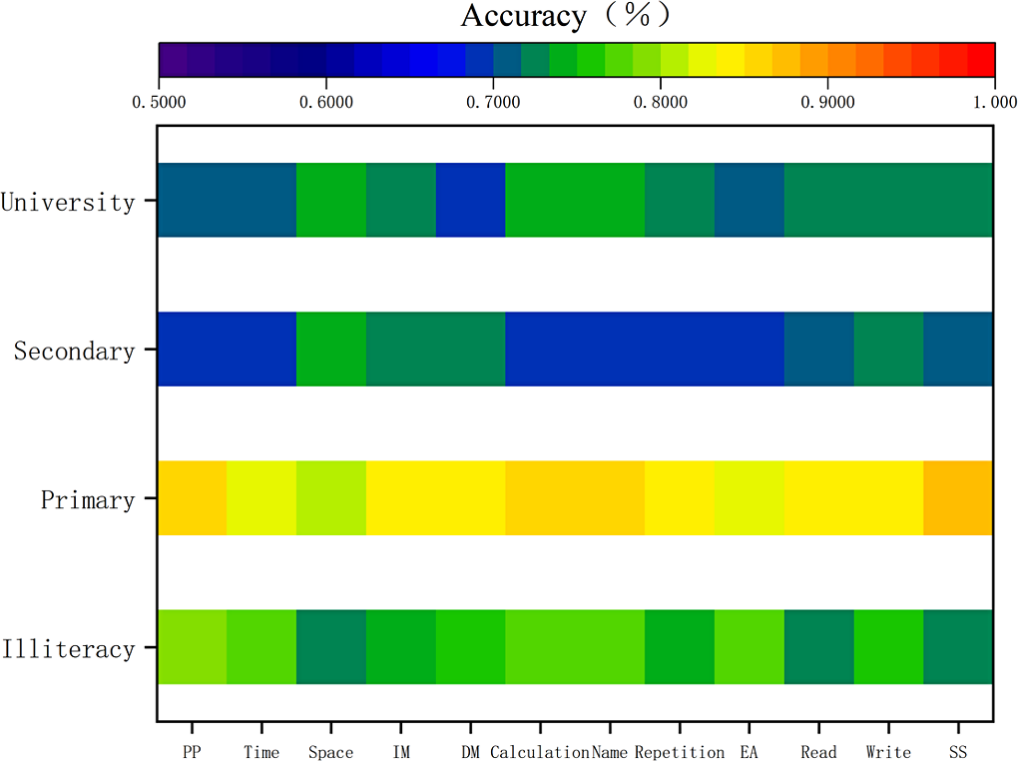
Heatmap visualization of feature contribution analysis. The color spectrum reflects prediction accuracy after item deletion. Cooler colors (blue and green) denote "critical features" (accuracy drop), while warmer colors (yellow and red) denote "neutral/interfering features" (accuracy stable/increase). The y-axis represents educational groups with the following sample sizes (N_patient/N_control): illiterate (60/48), primary school (53/52), secondary school (185/179), and university (106/129). DM: delayed memory; EA: execution ability; IM: immediate memory; PP: penmanship and praxis; SS: spatial structure.

To assess the contribution of individual MMSE items across educational groups, we conducted an item deletion experiment. The change in cross-validated accuracy (Δi) after removing each subitem was used to quantify its importance. A negative Δi (< -1.0) indicated a critical item whose removal impaired performance, while a positive Δi (> +0.5) indicated an interference item whose removal improved accuracy. The results of this analysis are visualized in Figure 2.

### Illiterate Group

In the illiterate cohort (baseline accuracy=78.83%), all subitems demonstrated positive contributions. The most influential items were spatial orientation (Δ=−6.58% postremoval), immediate memory (Δ=−3.85%), and writing (Δ=−2.86%). No interference items were identified.

### Primary School Group

In the primary school group (baseline accuracy=85.71%), spatial construction functioned as an interference factor (Δ =+0.95%), while calculation showed a negligible effect (Δ=0). Removal of immediate memory, delayed recall, reading, or writing reduced accuracy to 83.81% (Δ=−1.90%).

### Secondary School Group

In the secondary school group (baseline accuracy=69.78%), multiple interference factors were observed: spatial orientation (Δ=+3.57%), delayed recall (Δ=+3.02%), executive function (Δ=+2.19%), reading (Δ=+1.64%), immediate memory (Δ=+2.47%), writing (Δ=+0.20%), and spatial construction (Δ=+1.10%). Only temporal orientation (Δ=−0.83%) and calculation ability (Δ=−0.80%) demonstrated positive contributions.

### University Group

In the university group (baseline accuracy=71.49%), five interference items were identified: spatial orientation (Δ=+2.98%), calculation (Δ=+2.98%), immediate memory (Δ=+1.70%), naming (Δ=+1.70%), and delayed recall (Δ=+1.42%). Repetition (Δ=−0.57%) and reading (Δ=−0.30%) showed small positive contributions.

### Item Weight Analysis

On the basis of these contribution patterns, education-specific weighted coefficients were calculated for all MMSE subitems. The weighted diagnostic score was defined as follows:

$\text{S}\text{=}\sum_{t-1}^{11}\;(v_{i}\times w_{1})$

where S is the final weighted diagnostic score, v_i_ is the raw score of the i-th MMSE subitem, and w_i_ is the dynamic weighting coefficient assigned to the i-th subitem.

Classification was determined by comparing S to a group-specific classification threshold (T): S<T indicated cognitive impairment, and S≥T indicated normal cognition.

After normalization, the maximum possible weighted score was 60. Education-specific diagnostic thresholds were defined as follows: illiterate (*t*=30), primary (*t*=31), secondary (*t*=32), and university (*t*=33). As presented in Tables3  4, accuracy improved across all educational strata after weight calibration.

**Table 3.** Model performance and weighting coefficients for core cognitive domains by educational group.

Group	Prediction accuracy before weighting	Prediction accuracy after weighting	Orientation (time)	Orientation (space)	Memory (immediate)	Memory (delayed)	Attention and calculation
Illiterate (%）	79.83	86.08	1	3	2	2	1
Primary school (%）	85.71	88.16	2	3	2	2	1
Secondary school (%）	69.78	69.83	2	3	2	1	2
University (%）	71.49	73.62	1	3	2	2	2

**Table 4.** Weighting coefficients for language and visuospatial domains by educational group.

Group	Naming	Repetition	Execution ability	Reading	Writing	Spatial structure
Illiterate (%)	3	3	2	3	2	3
Primary school (%)	1	2	3	2	2	1
Secondary school (%)	2	2	2	1	1	1
University (%)	2	3	1	1	2	2

Table 5 summarizes the diagnostic performance before and after dynamic weighting, including accuracy, sensitivity, specificity, AUC, and the statistical significance of performance changes. The largest improvement occurred in the illiterate group (Δ=+7.25%), whereas the secondary school group showed the smallest improvement (Δ=+0.05%).

Weight distributions differed across education levels. For basic cognitive domains, spatial orientation weights were uniformly set to 3 across all groups, and immediate memory weights remained at 2. In the illiterate group, naming, repetition, and spatial construction received the highest weights (all=3). In the primary school group, temporal orientation was weighted at 2 and spatial construction was weighted at 1. In the secondary school group, executive function and calculation were weighted at 2, while spatial construction remained at 1. In the university group, executive function received the highest weight (3), calculation was weighted at 2, and spatial construction, reading, and writing each received a weight of 1.

**Table 5.** Comparison of diagnostic performance metrics and statistical significance before and after dynamic weighting.

Education group and metric	Before weighting	After weighting	Improvement (Δ)	*P* value
Illiterate			7.25%	.06
Accuracy (%)	78.83	86.08		
Sensitivity (%)	81.45	84.92		
Specificity (%)	76.21	87.24		
AUC^a^	0.835	0.912		
Primary school			2.45%	.03
Accuracy (%)	85.71	88.16		
Sensitivity (%)	86.10	88.54		
Specificity (%)	85.32	87.78		
AUC	0.894	0.928		
Secondary school			0.05%	.46
Accuracy (%)	69.78	69.83		
Sensitivity (%)	70.25	70.31		
Specificity (%)	69.31	69.35		
AUC	0.752	0.753		
University			2.13%	.18
Accuracy (%)	71.49	73.62		
Sensitivity (%)	72.15	74.88		
Specificity (%)	70.83	72.36		
AUC	0.768	0.795		

a AUC: area under the curve.

## Discussion

### Principal Findings

This study developed an education-sensitive optimization framework for the MMSE using a dynamic SVM-based weighting strategy. Across 812 education-stratified participants, the model demonstrated that cognitive subitems contribute differently to diagnostic prediction depending on years of formal education. Dynamic weighting enhanced the performance of unimodal MMSE classifiers, particularly in low-literacy groups, and reduced education-related diagnostic bias. These findings refine the diagnostic utility of condensed cognitive screening tools and support their broader applicability in resource-limited settings.

### Comparison With Prior Work

ML has been widely applied to cognitive assessment [32-35], yet existing studies typically use MMSE scores as features or labels without addressing education-driven measurement bias [36,37]. Prior condensed MMSE-only classifiers generally achieve accuracies of 72% to 85%, whereas multimodal MRI-, PET-, and biomarker-based models often exceed 90% [32,33]. Although our model remains unimodal, the dynamic weighting mechanism improved prediction by amplifying high-value signals and attenuating education-dependent interference, partially narrowing the performance gap with multimodal systems while sustaining low cost and operational simplicity.

Traditional MMSE optimization approaches—such as linear education corrections or standard ML models—lack the capacity to capture nonlinear, education-specific feature interactions [38]. In contrast, our framework integrates item deletion experiments with SVM-informed dynamic weighting to generate transparent, subgroup-adaptive coefficients. This approach offers an interpretable alternative to black box ensemble models and provides an effective mechanism for addressing item-level educational bias in cognitive assessment. A comprehensive comparison between the proposed dynamic weighted SVM model and existing cognitive assessment optimization strategies is presented in Table 6.

**Table 6.** Comparison of the proposed dynamic weighted Support Vector Machine (SVM) model with existing cognitive assessment optimization strategies.

Optimization strategy	Key methodology	Data requirements	Advantages	Limitations
Traditional linear correction (eg, MMSE^a^-E)	Linear regression and fixed point addition	MMSE scores+demographics	Simple calculation and clinically familiar	Ignored nonlinear “cognitive interference” and low precision
Standard machine learning (eg, RF^b^, ANN^c^)	Black box classification using raw scores	MMSE subitems or total scores	High classification accuracy	Low interpretability (“black box”) and hard to explain to clinicians
Multimodal fusion models	Deep learning (CNN^d^/RNN^e^) integration	MRI^f^/PET^g^ imaging+biomarkers+scale	Highest accuracy (>90%) and comprehensive pathology mapping	High cost, low accessibility in primary care, and complex deployment
This study (dynamic weighted SVM)	Nonlinear dynamic weighting via SVM	MMSE subitems only (low cost)	High interpretability (visible weights), education adaptive, and high accessibility	Accuracy is lower than multimodal models (feature limitation)

a MMSE: Mini-Mental State Examination.

b RF: random forest.

c ANN: artificial neural network.

d CNN: convolutional neural network.

e RNN: recurrent neural network.

f MRI: magnetic resonance imaging.

g PET: positron emission tomography.

### Interpretation of Education-Specific Patterns

Education-stratified analyses revealed distinct, cognitive reliance patterns. The illiterate group depended heavily on orientation and immediate memory, consistent with basic functional domains commonly preserved in low-literacy populations. The primary school group demonstrated changes in weighting for temporal orientation, whereas secondary school participants exhibited varied interference across multiple domains and minimal model improvement (*Δ*=0.05), suggesting high within-group heterogeneity. University-educated participants showed stronger reliance on executive functioning and calculation. These patterns align with prior evidence that cognitive performance becomes increasingly distributed and strategy dependent with higher educational attainment [39,40].

The dynamic weighting strategy provided incremental accuracy improvements across all educational cohorts while reducing false-positive rates in lower-education groups. Subitem deletion further allowed visualization of feature importance (Figure 2), conceptually analogous to Shapley Additive Explanations–based explainability, thereby enhancing the transparency of SVM decision processes. Clinical validation by senior rehabilitation physicians confirmed that weight distributions reflected plausible neurocognitive patterns.

External validation using an independent community dataset (n=314) demonstrated stable performance (overall accuracy: 82.48% and AUC: 0.88), with the highest accuracy observed in the illiterate group (88.89%). These results support the generalizability and robustness of the proposed dynamic weighting model and mitigate concerns about overfitting.

### Limitations

This study has several limitations. First, the sample was derived from a single geographic region, leading to educational imbalance across subgroups and potentially limiting generalizability. Broader multicenter sampling is needed. Second, demographic confounders beyond age and gender—such as socioeconomic status, occupational complexity, and urban-rural residence—were not available in the dataset, restricting the ability to fully disentangle education effects from related social determinants. Third, although the MMSE provides valuable screening utility, ceiling effects in highly educated individuals limit sensitivity to early cognitive decline. Integrating additional modalities, such as the Montreal Cognitive Assessment or imaging markers, may complement the proposed model. Finally, the SVM model relied on grid-search hyperparameter tuning; future research may explore AutoML-based optimization to improve efficiency and predictive performance.

### Conclusions

This study proposes a dynamic SVM-based weighting framework that enhances the diagnostic fairness of MMSE-based cognitive screening across diverse educational backgrounds. By quantifying item-level contributions and adapting subitem weights for each education group, the method addresses a longstanding source of measurement bias in cognitive assessment. The approach retains the accessibility and scalability of condensed cognitive screening tools while improving prediction accuracy and interpretability. These findings provide a practical foundation for developing equitable cognitive assessment strategies, particularly in resource-limited regions.

## Abbreviations

AUC: area under the curve
ML: machine learning
MMSE: Mini-Mental State Examination
MRI: magnetic resonance imaging
PET: positron emission tomography
SVM: support vector machine
